# Individual, social and physical environmental correlates of ‘never’ and ‘always’ cycling to school among 10 to 12 year old children living within a 3.0 km distance from school

**DOI:** 10.1186/1479-5868-9-142

**Published:** 2012-12-10

**Authors:** Fabian Ducheyne, Ilse De Bourdeaudhuij, Heleen Spittaels, Greet Cardon

**Affiliations:** 1 Department of Movement and Sports Sciences, Ghent University, Watersportlaan 2, 9000, Gent, Belgium

**Keywords:** Cycling, Children, Correlates, Distance

## Abstract

**Background:**

Cycling to school has been identified as an important target for increasing physical activity levels in children. However, knowledge about correlates of cycling to school is scarce as many studies did not make a distinction between walking and cycling to school. Moreover, correlates of cycling to school for those who live within a distance, that in theory would allow cycling to school, stay undiscovered. Therefore, this study examined individual, social and physical environmental correlates of never and always cycling to/from school among 10 to 12 year old Belgian children living within a 3.0 km distance from school.

**Methods:**

850 parents completed a questionnaire to assess personal, family, behavioral, cognitive, social and physical environmental factors related to the cycling behavior of their children. Parents indicated on a question matrix how many days a week their child (1) walked, (2) cycled, was (3) driven by car or (4) public transport to and from school during fall, winter and spring. Multivariate logistic regression analyses were conducted to examine the correlates.

**Results:**

Overall, 39.3% of children never cycled to school and 16.5% of children always cycled to school. Children with high levels of independent mobility and good cycling skills perceived by their parents were more likely to always cycle to school (resp. OR 1.06; 95% CI 1.04-1.15 and OR 1.08; 95% CI 1.01-1.16) and less likely to never cycle to school (resp. OR 0.84; 95% CI 0.78-0.91 and OR 0.77; 95% CI 0.7-0.84). Children with friends who encourage them to cycle to school were more likely to always cycle to school (OR 1.08; 95% CI 1.01-1.15) and less likely to never cycle to school (OR 0.9; 95% CI 0.83-1.0). In addition, children with parents who encourage them to cycle to school were less likely to never cycle to school (OR 0.78; 95% CI 0.7-0.87). Regarding the physical environmental factors, only neighborhood traffic safety was significantly associated with cycling: i.e., children were more likely to always cycle to school if neighborhood traffic was perceived as safe by their parents (OR 1.18; 95% CI 1.07-1.31).

**Conclusion:**

Individual, social and physical environmental factors were associated with children’s cycling behavior to/from school. However, the contribution of the physical environment is limited and highlights the fact that interventions for increasing cycling to school should not focus solely on the physical environment.

## Background

Physical activity has important health benefits in children. However, in many countries physical activity levels in children are assumed to be too low [1] and further declining [2]. Consequently many researchers have tried to identify target behaviors for increasing physical activity in children. Recently walking and cycling to school or “active commuting” have been identified as important targets for increasing physical activity levels in children by integrating physical activity into their daily routines [3].

Due to the increased popularity of active commuting to school as a target behavior, studies assessing correlates of active commuting to school by making use of an ecological approach [4] have expanded rapidly during the past five years. However, many studies did not make a distinction between walking and cycling to school, despite the fact that walking and cycling to school are two different behaviors. Both behaviors have a different impact on health [5] and their own characteristics and determinants. Furthermore, when it comes to interventions, most of the focus has been on walking to school. However, cycling to school enables active transportation to school from further distance, appears to be more energy intensive per unit of time [5], and has been shown to be related to higher levels of physical fitness compared to walking [6,7]. Yet in spite of these benefits, knowledge about correlates of cycling to school is scarce.

Only four studies could be located investigating correlates of cycling to school of which two were conducted in Europe [8-11]. Panter et al. [8] investigated correlates of cycling to school in the UK and found that social support from family and friends, parental perceptions of a safe neighborhood and route to school, and living in a high walkable neighborhood were positively associated with cycling to school. Moreover, this study was the only study that examined individual, social and physical environmental correlates in cycling to school simultaneously. A study conducted in Taiwan [9] investigated student’s perceptions of difficulties in cycling to school and found that being older or male, having the skills to safely ride a bicycle, live in a metropolitan area, and proper lighting and weather conditions were positively associated with cycling to school. Also, parental absence before or after school was found to be positively associated with cycling to school in the Netherlands [10]. Furthermore, Trapp et al. [11] reported that parental confidence in their child’s ability to cycle safely to school, parental perceptions of a safe neighborhood, parental inconvenience of car travel and no need to cross a busy road were positively associated with cycling to school in Australian primary school children. Additionally, in three of the four studies [8,10,11] living close to the school was positively associated with cycling to school.

Distance to school has been identified as one of the most important and consistent predictors of active commuting to school [12]. Distance to school is negatively associated with active commuting to school. British children were more likely to walk or cycle to school if their distance to school was less than 1 km [8]. Moreover, when distance increased from 750 m to 1.5 km, the proportion of Australian children performing 5 or more active trips dropped by one third, while the proportion of those performing no active trips doubled [12]. As distance to school is such a strong predictor, other factors that may predict cycling to school may be overlooked. Therefore, the investigation of other predictors for children living within a feasible distance for cycling to school is of interest. Recently, the identification of age-specific criterion distances to school [13-16], which represent feasible distances for children for active commuting to school, has been identified as an important strategy to remove distance as a confounding factor. Regarding correlates of cycling to school, there was only one study [7] who stratified the analyses by three categories based on the distance to school. However, the three distance categories used in this latter study were chosen based on the assumption that they would be appropriate for detecting possible transitions between walking and cycling and to maximize the numbers of children within each category. Studies investigating correlates of cycling to school for children living within a feasible cycling distance to school have not been conducted yet.

Understanding the different correlates of cycling to school may be particularly important during the transition from childhood into adolescence [13,14]. This critical period (10–12 years) is characterized by drop out from sports, increase of computer use and declining levels of physical activity [15,16]. However, during this transition children gain more autonomy and decision-making power regarding physical activity [17] and their independent mobility increases [18], as children get increased permission to cycle independently. This may result in increased cycling behaviors’, which may counter act the declining physical activity levels during the transition from childhood into adolescence. In addition, Cardon et al. [19], in press found that cycling to school at age 10 already strongly tracks into cycling to school at age 16, which highlights the importance of interventions promoting cycling to school at young age.

The aim of the present study was to examine individual, social and physical environmental correlates of cycling to/from school among Belgian 10 to 12 year old children using an ecological framework. Furthermore, only data of children living within a 3.0 km distance from school were analyzed. This 3.0 km criterion distance was found to be a feasible distance for Belgian children for cycling to school [20].

## Methods

### Sample and procedure

The present study was conducted during the winter (December-March) of 2010–2011. All data were obtained through the parents who were recruited through the schools of their child. In total, 50 primary schools were randomly selected in Flanders, Belgium and contacted by phone. From these schools, 22 principals agreed to let the 4^th^ to 6^th^ grade classes (children aged 10–12 year) of their school take part (response rate=44%). This resulted in 1550 parents that could be reached (one parent per child). Participating parents received a questionnaire through their child to fill out at home. Children were asked to hand the questionnaire to one of their parents and to bring the completed questionnaire back to school. A total of 1235 parents (response rate=80%) completed the questionnaire. Based on the 3.0 km criterion distance to school, data of 850 (69%) parents were analyzed. Ethical approval was granted, by the Ethics Committee of the Ghent University. Informed consent from all participating schools and parents was obtained.

### Questionnaire

The parental questionnaire was based on the literature [8,21-23] and supplemented with questions assessing specific cycling factors (e.g., parental attitude towards cycle training, parental perceived cycling skills of their child). The questionnaire was first pilot tested in a sample of 20 parents to improve the clarity of the questions. Test-retest reliability of the questionnaire was acceptable with ICC’s ranging from 0.31 to 0.97, indicating fair to perfect agreement [24], in press.

### Cycling behavior

Parents were asked about their child’s mode of transportation to school using the question matrix developed and found to be reliable by Bere and Bjørkelund [25]. In this matrix the parents indicated how many days a week their child (1) walked, (2) cycled, was (3) driven by car or (4) public transport to and from school during fall, winter and spring. Based on this matrix we were able to calculate the mean number of cycle trips per week to/from school [25].

### Factors

#### Demographics

Parents reported their child’s age and gender. To calculate body mass index (BMI), parents also reported the height and weight of their child.

#### Family factors

The number of children and number of cars in the household were assessed in the questionnaire. Parents reported their own and their partner’s highest level of education as a proxy measure of household economic status (low: no college or university education/high: having attended college or university). Within dual-parent households, only one parent needed to have attended college or university to be labeled as high. Furthermore, parents reported their family structure (single versus dual parent family).

#### Personal factors

Parents’ perceptions of the children’s motor competence and cycling skills were assessed. The questions were “in general, compared to other children of your child’s age, how would you rate your child’s motor competence?” and “in general, compared to other children of your child’s age, how would you rate your child’s cycling skills?”. A five-point Likert scale with response options ranging from “not good at all” to “excellent” was provided for both questions. Furthermore independent mobility of the child was assessed by the question ‘How far is your child allowed to leave home with the bicycle when he/she is alone?’. Response options were (1) not, (2) 0-500 m, (3) 500-1 km, (4) 1-3 km, (5) 3-5 km, (6) 5-10 km, and (7) more than 10 km.

#### Behavioral factors

Sports participation was evaluated by asking which sports (up to three) their child participates in during leisure time and how frequently (hours per week). The time spent in each sport was summed, and the average hours per day spent in sports participation were calculated. This part of the questionnaire was based on the Flemish Physical Activity Questionnaire, which is found to be reliable and valid [26]. Sedentary behavior was captured by asking how many hours per day their child spent watching TV, gaming and using a computer (which included chatting and internet surfing). Each screen time activity was separately assessed (hours per day). Test-retest reliability for the specific components of sedentary behavior was acceptable [27,28]. Parents also reported the number of hours their child spend reading (books, comic strips and magazines) during a usual week. Five response options were provided: (1) none, (2) less than 1 hour/week, (3) 1 to 6 hours per week, (4) 7 to 13 hours per week, and (5) more than 13 hours/week.

#### Parental attitudes

Parental attitude towards cycling to school and cycling to work were assessed with the questions “What do you think about cycling to school?” and “What do you think about cycling to work?”. Parents also reported their attitude towards sport and exercise with the question “What do you think about sport and exercise for your child?”. For the three questions a five-point Likert scale with response options ranging from “unimportant” to “very important” was provided. Also a question concerning the attitude towards cycling skills training programs was added in the questionnaire, namely “Do you think that a cycling skills training program could make your child safer in traffic?” (yes/no).

#### Risk factors

All parents were asked to indicate how many times a specific risk situation could happen on the way to school if their child would cycle to school. These risk situations were based on a questionnaire of Terence [29], which asked parents and children to assess the likelihood of risk situations that might afflict their journey to school.

#### Social environmental factors

Parents indicated agreement or disagreement with thirteen statements, that were either newly developed or adapted from existing scales [8,22], regarding their perceptions of their local neighborhood. Six statements referred to social support by family and friends, three statements referred to social support by the neighborhood, two statements referred to the social network of the child and another two statements looked at the neighborhood social cohesion.

#### Cognitive factors

Parents indicated how much they agreed or disagreed with four statements about their child’s habit of cycling to school. These four items were adapted from the scale of Verplanken [30] which measures habit strength. Furthermore, parents were asked about their agreement on three statements referring to the perceived behavior control of their child to accomplish cycling to school. These three statements were newly developed.

#### Physical environmental factors and route factors

The parent version of the ‘Neighborhood Environmental Walkability Survey for Youth’ (NEWS-Y) was used to assess potential cycling to school related environmental variables. This questionnaire is found to be reliable and valid in other settings [31]. The following aspects were assessed: a) residential density by three items; b) walking/cycling facilities by four items; c) maintenance of walking and cycling facilities by five items; d) connectivity by three items; e) accessibility by five items ; f) esthetic by four items; g) traffic safety by six items; and h) crime safety by four items. Five-points scales were used to standardize measurement levels for all variables studied. Furthermore, parents were asked about the route to school (eight statements), using “yes” or “no” response categories. The exact wording of the statements are shown in Table 1. In addition, parents reported the distance to school in kilometers.

**Table 1:** Individual, social and environmental correlates associated with cycling to/from school in Logistic regression models

	**Associations with never cycling to/from school**		**Associations with always cycling to/from school**	
	**Independent models N = 850**	**Fully adjusted model N = 850**	**Independent models N = 850**	**Fully adjusted model N = 850**
**Variables**	**OR (95% CI)**	**OR (95% CI)**	**OR (95% CI)**	**OR (95% CI)**
**Family factors**				
SES (ref. low SES)	1.0 (0.93-1.08)	NI	1.01 (0.96-1.07)	NI
Number of children in household (ref. no siblings)	0.94 (0.85-1.04)	NI	1.09 (1.02-1.17)*	1.05 (0.95-1.15)
Family structure (ref. dual)	1.15 (1.05-1.25)**	1.11 (1.02-1.2)*	0.93 (0.87-0.98)*	0.92 (0.86-0.98)**
**Personal factors**				
Independent mobility with the bicycle (ref. low)	0.74 (0.68-0.79)***	0.84 (0.78-0.91)***	1.15 (1.09-1.22)***	1.06 (1.0-1.13)*
Parental perceived motor competence of the child (ref. low)	0.95 (0.88-1.03)	NI	1.03 (0.97-1.09)	NI
Parental perceived biking skills of the child (ref. not good)	0.69 (1.63-0.76)***	0.77 (0.7-0.84)***	1.15 (1.07-1.23)***	1.08 (1.01-1.16)*
**Behavioral factors (ref. low)**				
Sports participation	0.95 (0.88-1.02)	NI	1.0 (0.95-1.05)	NI
TV-watching	0.99 (0.93-1.06)	NI	1.08 (1.03-1.14)**	1.09 (1.04-1.15)***
PC-using	1.01 (0.93-1.09)	NI	1.02 (0.97-1.07)	NI
Reading	0.98 (0.92-1.05)	NI	1.0 (0.95-1.05)	NI
**Attitudinal factors (ref. not important)**				
Attitude towards physical activity	0.94 (0.78-1.15)	NI	1.06 (0.92-1.22)	NI
Attitude towards cycling to school	0.78 (0.72-0.84)***	0.89 (0.81-0.97)**	1.13 (1.07-0.20)***	1.03 (0.97-1.1)
Attitude towards cycling to work	0.92 (0.85-0.99)**	1.04 (0.96-1.11)	1.01 (0.96-1.06)	NI
Attitude towards cycling training	1.06 (0.96-1.18)	NI	1.02 (0.95-1.09)	NI
**Risk factor (ref. low)**	1.12 (1.05-1.2)***	1.03 (0.96-1.1)	0.92 (0.88-0.97)**	0.96 (0.92-1.02)
**Social environmental factors (ref. disagree)**				
As parents we walk/bike along with our child to school	0.92 (0.86-0.99)*	0.95 (0.89-1.03)	0.93 (0.88-0.97)**	0.91 (0.87-0.96)***
Siblings often active commute to school	0.83 (0.77-0.89)***	0.99 (0.9-1.08)	1.14 (1.08-1.2)***	1.04 (0.97-1.11)
Friends often active commute to school	0.89 (0.83-0.96)**	1.0 (0.93-1.08)	1.05 (0.99-1.1)	NI
As parents, we encourage our child to actively commute to school	0.68 (0.63-0.73)***	0.78 (0.7-0.87)***	1.2 (1.13-1.26)***	1.0 (0.92-1.08)
Siblings encourage my child to actively commute to school	0.8 (0.75-0.86)***	1.0 (0.92-1.09)	1.19 (1.14-1.26)***	1.04 (0.98-1.11)
Friends encourage my child to actively commute to school	0.81 (0.75-0.88)***	0.9 (0.83-1.0)*	1.18 (1.12-1.24)***	1.08 (1.01-1.15)*
In my neighborhood many children active commute to school	0.9 (0.85-0.98)**	1.04 (0.96-1.12)	1.1 (1.05-1.16)***	1.02 (0.96-1.08)
In my neighborhood many parents active commute to work	0.9 (0.83-1.0)	NI	0.97 (0.92-1.02)	NI
In my neighborhood many parents walk/bike along with their child to school	1.01 (0.93-1.09)	NI	0.97 (0.92-1.02)	NI
Many peers of my child live in my neighborhood	0.93 (0.87-1.0)	NI	1.03 (0.98-1.08)	NI
People around here are willing to help their neighbors	0.95 (0.88-1.02)	NI	0.99 (0.94-1.04)	NI
This is a close knit neighborhood	1.0 (0.94-1.08)	NI	0.99 (0.94-1.05)	NI
My child often plays in the street with other kids in my area	0.9 (0.84-0.97)**	0.94 (0.88-1.02)	1.05 (1.0-1.10)	NI
**Cognitive factor (ref. low)**				
Habit of cycling to school	0.81 (0.76-0.88)***	1.02 (0.93-1.11)	1.25 (1.19-1.31)***	1.18 (1.11-1.25)***
-*Cycling to school is something my child does automatically*				
-*Cycling to school is something that belongs to my child*’*s daily routine*				
-*Cycling to school is something that my child typifies*				
-*Cycling to school is something my child has been doing for a long time*				
Perceived behavior control for cycling to school	0.82 (0.74-0.92)***	0.84 (0.75-0.93)**	1.01 (0.93-1.09)	NI
-*My child is able to bicycle to school on a regular base*				
-*It’s up to my child to decide if he/she bicycle to school*				
-*It’s up to the parents to decide if their child bicycles to school*				
**Physical environmental factors**				
Residential density (ref. low)	1.09 (1.01-1.16)*	1.02 (0.94-1.09)	1.0 (0.94-1.05)	NI
Walking and cycling facilities (ref. not good)	0.93 (0.83-1.05)	NI	0.99 (0.91-1.08)	NI
Connectivity (ref. low)	0.98 (0.9-1.05)	NI	0.98 (0.93-1.04)	NI
Esthetics (ref. not good)	0.97 (0.89-1.06)	NI	1.02 (0.96-1.08)	NI
Traffic safety (ref. not safe)	0.88 (0.77-1.0)*	0.98 (0.85-1.14)	1.19 (1.08-1.31)***	1.18 (1.07-1.31)***
Crime safety (ref. not safe)	0.97 (0.9-1.04)	NI	1.0 (0.95-1.05)	NI
**Route factors to school (ref. no)**				
Route along quiet roads	0.9 (0.83-0.97)**	1.09 (0.93-1.25)	1.07 (1.01-1.13)*	0.98 (0.89-1.09)
Route along busy roads	1.14 (1.05-1.23)***	1.08 (0.92-1.25)	0.94 (0.88-0.99)*	1.0 (0.9-1.12)
Route along roads with walking and cycling facilities	1.19 (1.11-1.27)***	1.18 (1.1-1.27)***	0.93 (0.88-0.98)**	0.92 (0.88-0.97)**
Route along roads with streetlights	0.96 (0.86-1.08)	NI	0.97 (0.90-1.05)	NI
Route along (a) road(s) with a steep incline	1.05 (0.9-1.22)	NI	0.95 (0.86-1.06)	NI
Route along a busy intersection	1.05 (0.97-1.14)	NI	0.96 (0.91-1.02)	NI
Route along the center of town	1.05 (0.97-1.14)	NI	0.97 (0.91-1.03)	NI
Route along the countryside	1.14 (0.81-0.97)**	0.95 (0.86-1.04)	0.99 (0.92-1.06)	NI

All analyses adjusted for age, gender, child BMI, household car access and distance to school.

*p<0.05, **p<0.01, ***p<0.001 (all bolded).

CI, confidence interval; OR, odds ratio; SES, socioeconomic status; NI, not included in the model.

### Statistical analyses

PASW Statistics 18 was used to describe the characteristics of the sample. Multivariate logistic regression analyses were conducted using MLwiN version 2.24. Multi-level modeling (two-level; participants-school) was applied to take clustering of participants in schools into account. Two regression analyses were conducted: one estimating the odds of never cycling to or from school (= 0 trips/week) and a second analysis estimating the odds of always cycling to or from school (= 10 trips/week). In both regression analyses, children who ‘never’ or ‘always’ cycled to school were compared with all other children. Furthermore, for each regression analyses two sets of models were created: one examining the effect of each variable on cycle frequency to school independently, while adjusting for the hypothesized confounding effect of age, gender, child BMI and household car access (= independent model) and a second model which fully adjusted for all variables included in the model (= fully adjusted model). Variables included in the second model needed to be statistically significant in the independent analyses. A continuous measure of distance was also included in the models to control for the confounding effect. Additionally, as one could hypothesize that the influences on cycling behavior may differ by age, we tested for interactions between the variables and cycling by children’s age (ref. young children). For the purpose of analysis, all five-points response scales were dichotomized. In the analyses no distinction was made between cycling to and cycling from school. To facilitate reading “cycling to/from school” was further referred to as “cycling to school”. All analyses were considered significant at p<0.05.

## Results

### Sample characteristics

Descriptive characteristics of the sample are presented in Table 2. In our sample, boys and girls were equally distributed. Children’s ages ranged from 8 to 13 years with a mean of 10.38 (SD=0.95) years. Eighty-nine percent of children were normal weight, and 64.1% of children had at least one parent with a bachelor’s degree or higher. Children lived, on average, 1.34 km (SD=0.83) from the school. Furthermore, 39.3% of children never cycled to school, while 16.5% of children always cycled to school.

**Table 2:** Background characteristics of the sample (n=850)

**Sex**	**%**
Boys	50.8
Girls	49.2
**Age**	**year**
Mean age (SD)	10.38 (0.95)
**BMI**	**%**
Normal	89
Overweight	9.6
Obese	1.4
**SES**	**%**
Low	35.9
High	64.1
**Distance to school**	**km**
Mean distance (SD)	1.34 (0.83)

(SD)= Standard deviation.

### Associations with never cycling to school (= 0 trips/week)

Table 1 presents which individual, social or physical environmental variables are associated with never cycling to school. Children from single-parent households were more likely to never cycle to school (OR=1.11; CI=1.02, 1.2) compared to children from dual-parent households. Furthermore, children with a high level of independent mobility (OR=0.84; CI=0.78, 0.91), with good cycling skills as perceived by their parents (OR=0.77; CI=0.7, 0.84), with friends encouragement (OR=0.9; CI=0.83, 1.0), with parental encouragement (OR=0.78; CI=0.7, 0.87) and a positive attitude of parents towards cycling to school (OR=0.89; CI=0.81, 0.97) had a decreased likelihood of never cycling to school. Further, children with a high degree of perceived behavior control for cycling to school had also a reduced likelihood of never cycling to school ((OR=0.84; CI=0.75, 0.93). In addition, children with a route to school along roads with walking and cycling facilities were more likely to never cycle to school (OR=1.18; CI=1.1, 1.27). Finally, no significant interaction effects were found between the variables and children’s age (results not shown).

### Associations with always cycling to school (= 10 trips/week)

Table 1 presents which individual, social or physical environmental variables are associated with always cycling to school. Children from single-parent households were less likely to always cycle to school (OR=0.92; CI=0.86, 0.98) compared with children from dual-parent households. Furthermore, children with a high level of independent mobility (OR=1.06; CI=1.0, 1.13), with good cycling skills as perceived by their parents (OR=1.08; CI=1.01, 1.16), with friends encouragement (OR=1.08; CI=1.01, 1.15), who watch a lot of TV (OR=1.09; CI=1.04, 1.15), and who are in the habit of cycling to school (OR=1.18; CI=1.11, 1.25) had an increased likelihood of always cycling to school. Further, children with parents cycling along to school had a reduced likelihood of always cycle to school (OR=0.91; CI=0.87, 0.96). In addition, children were more likely to always cycle to school if neighborhood traffic was perceived as safe by their parents (OR=1.18; CI=1.07, 1.31) and less likely to always cycle to school if they had a route along roads with walking and cycling facilities (OR=0.92; CI=0.88, 0.97). Finally, the only significant interaction was found between independent mobility with the bicycle and children’s age (OR=1.09; CI=1.01, 1.16). Older children with a high level of independent mobility were more likely to always cycle to school compared with younger children with a high level of independent mobility. For all other variables no significant interactions were found (results not shown).

## Discussion

This study examined the associations between individual, social and physical environmental factors and children’s cycling to school and is one of the first studies to take an age-specific criterion distance for cycling to school into account. We found evidence that in children living within a feasible distance to school individual, social and physical environmental factors were associated with cycling to school. However, it should be noted that neighborhood traffic safety was the only physical environmental factor associated with cycling to school, indicating that the contribution of the physical environment to cycling to school was limited.

In the present study, children from single-parent households were more likely to never cycle to school and to cycle to school irregularly. This finding is in contrast with other studies [12,23] who found that family structure was not significantly associated with active commuting to school. However, in these latter studies no distinction was made between walking and cycling to school, which may explain the different results. Consequently, no univocal decision may be reached, indicating that more research is required on the contribution of family structure to cycling to school.

Consistent with other studies [12,32,33] higher levels of independent mobility were related to more cycling to school. Children who were allowed to cycle alone a certain distance from home were more likely to always cycle to school and less likely to never cycle to school. A possible explanation may be that at this age parents’ direct involvement in active commuting to school with their child is less common [33]. In addition, the significant interaction between independent mobility and children’s age, predicting the odds of always cycling to school, indicated that the difference in children’s cycling behavior between children with low and high levels of independent mobility is more pronounced in older children. Furthermore, children whose biking skills were perceived as good by their parents were less likely to never cycle to school and more likely to always cycle to school. This finding is in line with the study of Trapp et al. [11] who found that parental confidence in their child’s ability to cycle to school played a mediating role in the association between perceived safety and cycling to school. This study also highlighted the need for educational programs focusing on the development of children’s cycling skills. Our study result underpin this need. Additionally, the fact that reported cycling skills, perceived by the parents, were associated with cycling to school leads us to believe that parents do take the cycling skills of their child into account when deciding to allow their child to cycle to school or not.

An association between parental attitude towards cycling to school and never cycling to school was observed. Similarly, in the study of McMillan [34] caregivers who valued the social interaction for their child on the trip to school had children that were more likely to active commute to school. Furthermore, children with a high degree of perceived (by their parents) behavior control for cycling to school were less likely to never cycle to school, whereas children who are in the habit of cycling to school were more likely to always cycle to school. These findings are in line with a study of Lemieux and Godin [35] investigating how well cognitive variables predict active commuting to school. It should be noted that in our study child’s perceived behavior control and habit of cycling to school were reported by parents. However, these results still show that cognitive variables are important predictors of cycling to school, which highlights the importance of cognitions within interventions promoting cycling to school.

Consistent with other studies [8,36,37], parental and friend encouragement were also important correlates of cycling to school. Children with friends who encourage them to cycle to school were more likely to always cycle to school and less likely to never cycle to school. In addition, children with parents who encourage them to cycle to school were less likely to never cycle to school. Based on these findings it seems that both sources of social support are important for cycling to school and that especially support from friends is needed to always cycle to school. Furthermore, we found that children were less likely to always cycle to school when parents cycle along with their child. A possible explanation could be that parents who cycle along with their child to school find the trip to school unsafe or have some doubts about the cycling skills of their child. So these parents probably won’t let their child cycle alone to school if they are unable to cycle along which increases the probability that their child becomes an irregular instead of a regular cycler.

Children were more likely to always cycle to school if neighborhood traffic was perceived as safe by their parents. The observation that traffic safety contributed to the prediction of cycling to school is in agreement with other studies [8,21,23,34,38]. Consequently, efforts like driver education, traffic calming and separate bicycle facilities seem of interest. Remarkably, neighborhood traffic safety was the only environmental factor associated with always cycling to school and no environmental factors were associated with never cycling to school. This finding suggests that the contribution of the physical environment to cycling to school within a criterion distance of 3.0 km from school is limited and highlights the fact that interventions for increasing cycling to school should not focus solely on the physical environment, which supports the view of other studies [35,38]. Furthermore, the present study found that children were more likely to never cycle and less likely to always cycle to school if their route to school was along roads with walking and cycling facilities. This is rather counter-intuitive, since one would expect the opposite. However, roads in Belgium that are equipped with walking and cycling facilities are usually quite busy. Busy roads have been identified as important barriers for active commuting to school [3,23,39].

One of the major strengths of this study is the incorporation of an age-specific criterion distance, which represent a feasible distance for children for cycling to school. Furthermore, an ecological approach was used to identify correlates of cycling to school. Additionally, a distinction was made between correlates of never cycling to school and correlates of always cycling to school. Finally, data were collected in a large sample. Several limitations of this study must be considered. Our data are cross-sectional in nature, indicating that causal relationships cannot be drawn. Further, generalization of this study is limited by the nature of the sample comprising children of the 4^th^ to 6^th^ grade only. However, this narrow age range was chosen in order to obtain a homogenous study population. Furthermore, this study relies only on parents’ reports as children’s perceptions were not measured. Additionally, no objective measures of the built environment were assessed. Notwithstanding this latter limitation, the environmental questions used in the questionnaire have been validated [31].

## Conclusion

In summary, several individual, social and physical environmental factors were associated with cycling to school for children living within a 3.0 km distance from school. Our results suggest that creating a positive attitude of parents towards cycling to school and teaching children the basic cycling skills are important strategies that should be taken into account when promoting cycling to school among children living within 3.0 km distance from school. Furthermore, encouraging social support by parents and friends, developing perceived behavioral control and habituation should all be considered as intervention goals for cycling to school. Since neighborhood traffic safety was the only environmental factor associated with cycling to school, interventions promoting cycling to school among children living within 3.0 km distance from school should not focus solely on the physical environment. Consequently, a multi-factorial intervention including personal, cognitive, social and physical environmental aspects might be a promising strategy for the promotion of cycling to/from school.

## Abbreviations

SES: Socioeconomic status; BMI: Body mass index; SD: Standard deviation; ICC: Intraclass correlation coefficient; OR: Odds ratio; 95% CI: 95 percent confidence interval.

## Competing interests

The authors declare that they have no competing interests.

## Authors’ contribution

FD and GC conceived and designed the study. FD carried out data analyses, interpretation and drafted the first outline of the paper. IDB, HS and GC made substantial contributions to interpretation of the data and revised the manuscript critically for important intellectual content. All authors read and approved the final manuscript.

## Acknowledgements

The authors want to thank Sara De Lepeleere and Winnie Thewissen for their assistance in data collection. This research was supported by the Life line campaign of the Research Foundation - Flanders (FWO) FWO B10823/02/03.
